# Localised crystal‐storing histiocytosis in a classical Hodgkin lymphoma patient post allogeneic transplant

**DOI:** 10.1002/jha2.780

**Published:** 2023-09-22

**Authors:** Carol Kwon, Christopher G. Mullen, Jennifer Buxton, Hadil AbuArqoub

**Affiliations:** ^1^ Department of Pathology, Western General Hospital NHS Lothian Edinburgh UK; ^2^ Department of Haematology, Western General Hospital NHS Lothian Edinburgh UK

1

A 31‐year‐old male with Hodgkin lymphoma, treated with pembrolizumab and sibling‐matched allogeneic transplant, attended clinic 2 years after consolidation therapy with a history of sudden onset, atraumatic right posterior hip pain. There was no neurological compromise and no palpable masses, although pain was reproducible on forced flexion of his right hip. Computed tomography (CT) of his hip and spine was unremarkable, and the patient underwent further imaging. Positron emission tomography (PET)–CT demonstrated a 13 mm rounded focus, standard uptake value maximum 10.6, adjacent to the right lateral recess and extending into the exit foramina at the L2 level, concerning relapsed disease. This abnormality was also noted on magnetic resonance imaging of his lumbar spine, although only after expert neuro‐radiological review.

Given the unusual site and lack of PET‐positive disease elsewhere, the patient underwent a neurosurgical biopsy of the mass and L2 decompression, which unfortunately did not alleviate his symptoms. The biopsy demonstrated diffuse sheets of polyglonal histiocytes with indistinct borders and abundant eosinophilic cytoplasm (Figure 1, top left, 40× magnification). Many of the histiocytes contained rhomboid refractile but non‐polarisable cytoplasmic crystals. These cells were diffusely positive for the pan‐macrophage marker CD68, suggesting crystal‐storing histiocytosis (CSH) (Figure 1, top right, 40× magnification). CD30/15^+^ Hodgkin and Reed‐Sternberg cells were not seen, refuting relapsed Hodgkin lymphoma. Small numbers of CD20^+^ B cells and CD3^+^ T cells were also identified.

**FIGURE 1:**
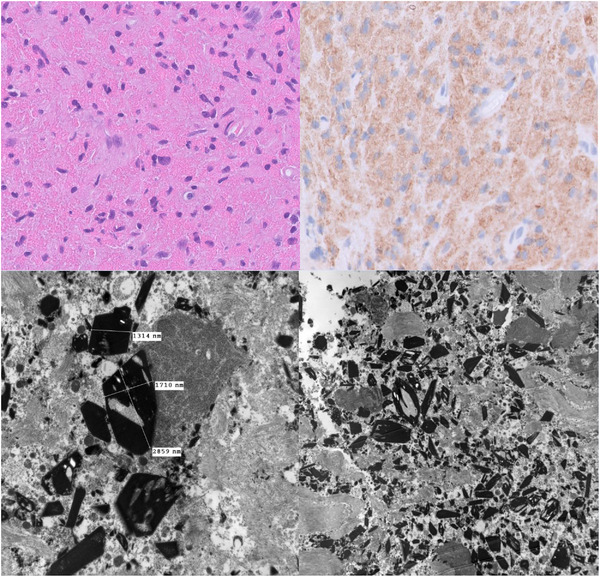
Left: Electron microscopy image showing crystals at 15,000× magnification. Right: Electron microscopy image showing crystals at 5000× magnification.

Electron microscopy performed on formalin‐fixed paraffin‐embedded tissue showed numerous intracellular rhomboid solid and basket‐weave/reticulated crystals measuring up to 2859 nm in maximum dimension (Figure 1, top left, H&E, 40× magnification; Figure 1, top right, anti‐CD68, 40× magnification; Figure 1, bottom left, 1500× magnification; Figure 1, bottom right, 5000× magnification).

The patient was commenced on corticosteroids, which resulted in rapid resolution of his pain, although there was a recrudescence on stopping.

CSH is a rare benign disorder characterised by the accumulation of non‐neoplastic histiocytes laden with intra‐lysosomal eosinophilic crystals. The crystals are most often composed of immunoglobulin fragments and this condition is usually associated with an underlying lymphoproliferative or plasma cell disorder. There is no established association between CSH and Hodgkin lymphoma in the literature. In our case, protein electrophoresis did not detect a paraprotein and serum free light chain ratio was normal (*κ* 8.1 mg/L, *λ* 8.2 mg/L, ratio 0.99), excluding an associated monoclonal gammopathy. A minority of cases may be seen with autoimmune disorders, inflammatory disorders and drug‐induced disorders, for example, clofamazine [1].

## AUTHOR CONTRIBUTIONS

Christopher G. Mullen and Jennifer Buxton wrote the clinical vignette with further input from Carol Kwon and Hadil AbuArqoub with respect to histological findings and electron microscopy findings. Carol Kwon took the electron microscopy photographs with the help of Fiona Young. All authors viewed and agreed on the final draft of this manuscript.

## CONFLICT OF INTEREST STATEMENT

The authors declare they have no conflicts of interest.

## FUNDING INFORMATION

The authors received no specific funding for this work.

## ETHICS STATEMENT

We did not seek local ethical approval for this publication but informed patient consent was obtained.

## Data Availability

Data sharing is not applicable to this article as no new data were created or analyzed in this study.
